# Development and application of an indirect enzyme-linked immunosorbent assay based on recombinant capsid protein for the detection of mink circovirus infection

**DOI:** 10.1186/s12917-018-1337-z

**Published:** 2018-01-26

**Authors:** Junwei Ge, Xingyang Cui, Yunjia Shi, Lili Zhao, Chengwei Wei, Shanshan Wen, Shuang Xia, Hongyan Chen

**Affiliations:** 1 College of Veterinary Medicine, Northeast Agricultural University, Harbin, 150030 China; 2 Laboratory Animal and Comparative Medicine Unit, Harbin Veterinary Research Institute, The Chinese Academy of Agricultural Sciences, No. 678 Haping Rd, Harbin, 150069 China; 3 Northeastern Science Inspection Station, China Ministry of Agriculture Key Laboratory of Animal Pathogen Biology, Harbin, 150030 China

**Keywords:** Mink circovirus, Capsid protein, Prokaryotic expression, Indirect ELISA, Serological survey

## Abstract

**Background:**

Mink circovirus (MiCV) is a newly discovered pathogen associated with mink diarrhea. The prevalence and economic importance of this virus remain poorly understood, and no specific serological assay has been developed for the diagnosis of MiCV infection.

**Results:**

In this study, a recombinant capsid protein antigen expressed in *Escherichia coli* was utilized to establish an indirect enzyme-linked immunosorbent assay (iELISA). Results revealed that the assay had no cross-reactivity with other related pathogens, and the respective sensitivity and specificity of the proposed iELISA were 92.31% and 91.67% compared with those obtained of Western blot on 138 serum samples from minks. The correlation coefficient between iELISA and Western blot was 0.838 (*p* > 0.05). iELISA was applied to detect MiCV antibodies in 683 clinical serum samples from different farms from the major mink industry province in China, and 21 of 24 farms with 163 of 683 (23.87%) individuals were tested positive for MiCV antibodies. The positive rates of each of the 21 flocks ranged from 2.33% to 73.68%.

**Conclusions:**

These results indicated that iELISA was a sensitive and specific method suitable for the large-scale detection of MiCV infections in mink. This study provided an effective method for the serological diagnosis and positive rate investigation of MiCV infection.

## Background

Mink circovirus (MiCV) is a newly discovered virus originally reported in 26 six- to ten-month-old minks with diarrhea from three farms in Dalian, China [1]. MiCV is a member of *Circovirus* belonging to the *Circoviridae* family. MiCVs are single-stranded circular DNA viruses with a genome of 1753 nucleotides, which contain two major open reading frames (ORF). One is ORF1, which encodes a replicase-associated protein (rep) required for viral replication. The other is ORF2, which encodes proteins that form the viral capsid (cap) and participate in host immune responses. Circoviruses have been identified in numerous species associated with various clinical disorders [2, 3], including asymptomatic infections and lethal diseases [4, 5]. Circovirus infections are also related to lymphoid depletion and immunosuppression, which likely increase the severity of secondary infection [6].

Epidemiological studies have provided evidence that MiCV is endemic in some mink farms in China [7]. However, the pathogenic role of MiCV in single or polymicrobial infections is unclear, and the prevalence and economic importance of this virus remain unknown. Therefore, a rapid and sensitive diagnosis assay or tool of the virus is essential for disease management and epidemiological surveillance.

Conventional Polymerase Chain Reaction (PCR) can detect MiCV [8]. Although PCR has been used to identify MiCV infections, other methods that can detect virus-specific antibodies in serum are more convenient for large-scale epidemiological investigations. Serological methods for the identification of antibodies against MiCV infection in mink farms have yet to be reported. Antigens based on virus isolation for MiCV diagnosis have yet to be prepared because there is no report about cell system to culture MiCV.

Our study developed a sensitive, specific, and convenient indirect enzyme-linked immunosorbent assay (iELISA) utilizing the recombinant capsid protein (rCap) of MiCV expressed in *E. coli* as a coating antigen. The proposed iELISA was strongly immunoreactive to MiCV-specific serum and had no cross-reactivity with other related pathogens. This assay was validated by comparing the obtained results with those of Western blot. The applications of the proposed assay for clinical detection were also described.

## Methods

### Blood samples

Rehabilitation sera collected from a MiCV infection case were used as MiCV-positive mink sera. This infection caused acute gastroenteritis, and the clinical signs observed in the mink included severe diarrhea, vomiting, lethargy, anorexia, dehydration, and rough fur. Common enteric bacterial, protozoan, and viral pathogens, including *Salmonella* spp*.*, *Clostridium* spp., *Campylobacter* spp., Shiga toxin-producing *E. coli*, enterotoxigenic *E. coli*, *Coccidium* spp., *Cryptosporidium* spp*.*, mink aleutian disease virus (AMDV), canine distemper virus (CDV), mink enteritis virus (MEV), caliciviruses, rotaviruses, coronaviruses, and astroviruses, were excluded as causative agents, and MiCV was subsequently identified as the possible cause of the disease.

Sixty healthy minks were sampled from a MiCV-free farm to represent negative control mink sera. The MiCV-free farm was selected on the basis of prior negative testing with PCR once a year for the last 3 years.

A total of 138 serum samples were selected blindly from a pool of sera collected from healthy, diseased, or dead minks in more than 30 mink farms in Heilongjiang and submitted in 2008–2012. These samples were then tested and compared with ELISA tests. All of the serum samples were stored at −20 °C until use.

This study was performed in accordance with the recommendations in the Guide for the Care and Use of Laboratory Animals of the Ministry of Health, China. Prior to experiments, the protocol of the current study was reviewed and approved by the Institutional Animal Care and Use Committee of Northeast Agricultural University (approved protocol number 2014-SRM-24).

### Expression, purification, and identification of the rCap protein

The MiCV HEB15 strain in this study was obtained previously by our team (GenBank accession No. KX268345) and used as the template in our current study. The gene encoding the Cap protein was amplified from the total viral DNA through PCR with the forward primer 5'-CGGGATCCGGCGGTTACAGATGGCG-3' and the reverse primer 5'-ACCTCGAGAGTTTGCTTTGGGA-3'. The amplified cap gene was subcloned into the *Bam*HI and *Xho*I sites of the prokaryotic expression vector pET-32a in a frame with an amino terminal six-histidine tag. Recombinant plasmids designated as pET32a–cap were transformed into *E. coli* Rossetta cells to express His-tagged rCap. pET32a–cap was sequenced with commercial primers (T7 and S tag) by Comate Biotechnology Company (Changchun, China). rCap was purified using a Ni-Agarose 6× His-tagged protein purification kit (Qiagen, German) according to the manufacturer’s instructions. rCap was identified through Western blot by utilizing convalescent sera from minks infected with MiCV or healthy mink sera as primary antibodies. Horseradish peroxidase-conjugated protein A/G (Thermo Scientific) was set as a detection reagent. Similarly, rCap was also identified through Western blot analysis by applying HRP-conjugated mouse anti-His MAb (Sigma, USA) as antibodies.

### Western blot analysis for the serum samples

Purified rCap protein and pET-32a vector protein as controls were separated on an SDS–polyacrylamide gel, and the samples were transferred to pure nitrocellulose blotting membranes (Pall Corporation, USA) for Western blot. These membranes were blocked with 5% skimmed milk–PBS for 2 h at 37 °C, then incubated for 2 h at 37 °C with serum samples diluted in 1:50, washed thrice with PBS–T (0.05% Tween 20 dissolved in PBS), and further incubated for 1 h with horseradish peroxidase-conjugated protein A/G. After the membranes were washed again with PBS–T, the bands were detected using DAB reagents (Biotopped, Beijing, China).

### ELISA procedure

An ELISA based on a recombinant Cap antigen was conducted according to a previously published protocol [9, 10]. In brief, 96-well polystyrene immunoplates (Jet Bio-Filtration, Guangzhou, China) were coated with 100 μl of rCap protein in 0.05 M bicarbonate/carbonate buffer (pH 9.6) overnight at 4 °C. The wells were then washed thrice with PBS (pH 7.4) containing 0.05% Tween 20 (PBS–T), and the plates were blocked with 1% BSA for 1 h at 37 °C. Three additional PBS–T washes were carried out, the serum samples were diluted in PBS (pH 7.4) containing 5% skim milk, 100 μl of the diluted samples was added to the wells, and the plates were incubated for 3 h at 37 °C with each serum sample in duplicate. The plates were washed with PBS–T before the detection reagent solution (horseradish peroxidase-conjugated protein A/G) diluted at 1:10,000 in PBS was added to each well, and the plates were incubated again for 60 min at 37 °C. The plates were washed thrice, 100 μl of TMB diluted in substrate buffer was added to each well, and the plates were incubated for 30 min at 37 °C. Finally, the reactions were stopped with 2 mol/L H_2_SO_4_, and optical density (OD) was read at 450 nm with a microplate reader (Bio-Tek Instruments, USA).

The optimum concentrations of coating antigen, serum dilution, and horseradish peroxidase-conjugated protein A/G antibody were determined through checkerboard serial-dilution analysis.

In brief, rCap was serially diluted twofold from 4 μg/ml to 0.125 μg/ml in 0.05 mol/L Na bicarbonate/carbonate buffer. MiCV-positive and MiCV-negative control mink sera were also serially diluted twofold from 1:25–1:400 and used to optimize rCap ELISA. After the optimal antigen concentration and serum dilutions were established, checkerboard titrations were performed to identify the optimal working dilutions of the conjugate. The conjugate was added to the plate at ratios of 1:10,000, 1:20,000, 1:40,000, and 1:80,000 to determine the optimal detection antibody dilution for this ELISA. The conditions that yielded the highest OD450 ratio between positive control and negative control sera (P/N value) were considered optimal.

### Determination of the cut-off value for iELISA

Negative serum control samples from 60 clinically healthy minks obtained from three farms without a history of MiCV and confirmed negative for MiCV antibodies through Western blot were diluted at the optimal dilution and assayed. The cut-off value was determined by calculating the mean OD of the negative samples in Western blot (*n =* 60) plus 3 standard deviation (SD).

### Evaluation of iELISA specificity

To evaluate the potential cross-reactivity of iELISA, we used the sera from the healthy minks as negative controls and the sera extracted from minks positive to canine adenovirus type 2 (CAV-2), AMDV, MEV, pseudorabies virus (PRV) and CDV or positive to *E. coli*, *Pasteurella multocida*, and *Pseudomonas aeruginosa*. These samples were examined in terms of reactivity in iELISA and were assayed in duplicate.

### Determination of the reproducibility of the assay

To determine the reproducibility of iELISA, we selected six serum samples from minks (three Western blot-positive samples and three Western blot-negative samples). To assess the intra-assay (within-plate) reproducibility, we ran six replicates of each sample on the same plate. For inter-assay (between-run) reproducibility, three replicates of each sample were run in different plates. Mean OD, SD, and coefficient of variation (CV) were calculated.

### Comparison of iELISA with western blot

To investigate the specificity and sensitivity of iELISA, we examined 138 serum samples collected from minks in Heilongjiang through iELISA and Western blot and calculated the correlation between these methods.

### Application of iELISA for the detection of MiCV infection

To estimate the positive rates of MiCV in the mink population, we randomly collected serum samples (*n* = 683) from 24 farms in Heilongjiang, Jilin, Shandong, Hebei, and Liaoning in China in 2014–2016. The iELISA was applied to detect these serum samples and to obtain antibody titers for any sample by serially diluting the samples in twofold.

## Results

### Expression and purification of rCap protein

**Fig. 1:**
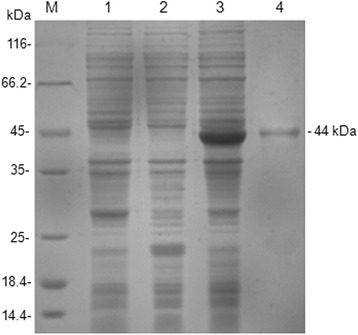
SDS-PAGE analysis of the rCap fusion protein. Lane M: protein molecular weight marker; Lane 1: Rosetta control; Lane 2: pET32a vector control; Lane 3: pET32a–cap bacterial lysate; Lane 4: Purified fusion protein

**Fig. 2:**
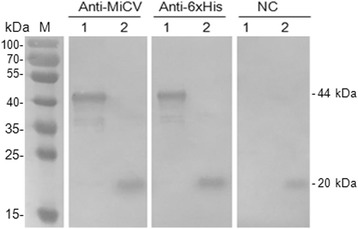
Western blot was performed with MiCV positive sera (Anti-MiCV), Anti-6xHis HRP conjugated (Anti-6xHis) or MiCV negative sera control (NC). Lane M: protein molecular weight marker; Lane 1: Purified protein rCap; Lane 2: pET32a vector control

The truncated MiCV capsid gene was amplified through PCR directly from the feces of the infected mink, and an expected band with a size of 600 bp was obtained. This gene was then cloned into the pET-32a expression vector. The construction of recombinant pET-32a-cap plasmid was confirmed through double digestion with *Bam*HI and *Xho*I and sequence analysis. The insert yielded 99% nucleotide identities with MiCV DL13 (GenBank accession no. NC_023885.1) and Hebei13 (GenBank accession no. KM586846.1). The recombinant plasmid pET-32a-cap was transformed into *E. coli* strain Rossetta (DE3). After induction, the recombinant protein (rCap) was expressed as an insoluble 44 kDa His-tagged fusion protein corresponding to the expected size. The fusion protein was purified using a Ni-NTA His Bind resin column and identified through SDS–PAGE analysis (Fig. 1). The antigenicity of the recombinant rCap protein was further confirmed through Western blot analysis involving positive serum samples and anti-His-tag monoclonal antibody (Fig. 2). The results indicated that only the 44 kDa rCap protein reacted strongly with the positive sera in Western blot, whereas the pET-32a vector protein did not react with the positive sera. The data revealed that the rCap protein exhibited a good immune reaction with a specific antibody to MiCV. This result indicated that the antigenicity of rCap protein was confirmed, and this protein antigen could be used to detect specific antibodies against MiCV. The purified rCap protein was utilized as a coating antigen in iELISA.

### Development of the rCap ELISA

Checkboard ELISAs were applied to determine the optimal coating antigen concentration and the optimal serum dilution, which were 0.5 μg/ml and 1:200, respectively (Table 1). After the optimal coating antigen concentration and the optimal serum dilution were set, other factors in rCap iELISA were optimized.

**Table 1:** Ratio of MiCV-positive serum to MiCV-negative serum for the optimization of assay conditions for the ELISA against recombinant rCap antigen

Serum dilution	Concentration of rCap antigen (μg/well)					
	4	2	1	0.5	0.25	0.125
1:25(+)	0.806	1.056	1.048	0.969	0.89	0.742
1:25(−)	0.108	0.166	0.16	0.153	0.133	0.104
1:50(+)	0.876	1.175	1.044	0.967	0.888	0.78
1:50(−)	0.175	0.116	0.144	0.12	0.116	0.09
1:100(+)	0.753	1.078	1.063	0.964	0.895	0.737
1:100(−)	0.096	0.121	0.129	0.1	0.114	0.089
1:200(+)	0.971	1.119	1.065	**1.004**	0.801	0.767
1:200(−)	0.113	0.127	0.15	**0.108**	0.097	0.088
1:400(+)	0.681	0.724	0.807	0.779	0.821	0.638
1:400(−)	0.094	0.068	0.111	0.083	0.083	0.068

Optimum values with the best positive-to-negative ratio are highlighted

We found that 1% BSA was the optimum blocking buffer for this assay, resulting in the highest P/N value among the tested blocking solutions. The optimal working dilution of the conjugate was 1:10,000. The incubation periods for the serum samples or the detection reagents respectively set at 90 or 60 min were observed to be the optimum.

To set a cutoff value for the rCap iELISA, 60 sera tested negative by Western blot were examined. The mean ± SD OD450 value for those sera in the rCap iELISA was 0.229561 ± 0.027542, which gave a cutoff value of 0.312 (mean + 3SD) for this assay. Therefore, serum samples with an OD450 > 0.312 in this assay were regarded as positive for MiCV.

No cross-reactions were detected by iELISA involving the antisera against the related pathogens, such as CAV-2, AMDV, MEV, PRV and CDV, *E. coli*, *P. multocida*, and *P. aeruginosa*, and the OD values ranged from 0.208 to 0.098 (Table 2). The data indicated that the developed iELISA exhibited good specificity for MiCV antibody detection.

**Table 2:** Results of cross test of indirect ELISA

Serum sample	Value of OD_450_
MiCV(+)	1.451
MiCV(−)	0.137
Blank control	0.109
AMDV	0.121
MEV	0.154
CDV	0.161
PRV	0.098
CAV-2	0.135
*Escherichia coli*	0.113
*Pasteurella multocida*	0.201
*Pseudomonas aeruginosa*	0.208

### Determination of assay reproducibility

**Fig. 3:**
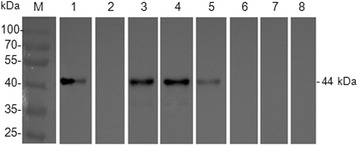
Western blot analysis of the rCap fusion protein. Lane M: protein molecular weight marker; Lane 1:Positive serum control; Lane 2: Negative serum control; Lane 3: Serum 1;Lane 4: Serum 2; Lane 5: Serum 3; Lane 6: Serum 4; Lane 7: Serum 5; Lane 8: Serum 6

The reproducibility of iELISA was determined by comparing CVs of each mink serum sample. The intra-assay CVs of three serum samples and three negative serum samples determined through Western blot (Fig. 3) and iELISA ranged from 2.15% to 6.23%, with a median of 4.295%, whereas the inter-assay CV of these samples varied from 2.40% to 8.02%, with a median of 5.335%. These data demonstrated that iELISA was repeatable, yielding acceptably low variation levels (Table 3).

**Table 3:** Repeatability and reproducibility analysis of indirect ELISA

Sample	Intra-assay(OD450)						Inter-assay(OD450)					
	1	2	3	Mean	SD	CV%	1	2	3	Mean	SD	CV%
1	0.382	0.401	0.432	0.405	0.025	6.23	0.386	0.413	0.411	0.403	0.015	3.73
2	0.641	0.664	0.594	0.633	0.036	5.64	0.604	0.656	0.693	0.651	0.045	6.87
3	0.471	0.487	0.468	0.475	0.010	2.15	0.459	0.466	0.481	0.469	0.011	2.40
4	0.282	0.271	0.256	0.270	0.013	4.84	0.248	0.281	0.243	0.257	0.021	8.02
5	0.244	0.261	0.252	0.252	0.009	3.37	0.253	0.258	0.225	0.245	0.018	7.25
6	0.180	0.194	0.188	0.187	0.007	3.75	0.182	0.195	0.194	0.190	0.007	3.80

### Comparison of iELISA with western blot

A total of 138 serum samples was investigated by iELISA, and the results were compared with those of Western blot. Of the 138 samples analyzed, 78 serum samples were detected as positive and 60 were identified as negative by Western blot. Furthermore, 77 serum samples were determined as positive and 61 samples were classified as positive by iELISA. Our findings also indicated that 72 were positive and 55 were negative by both assays. However, 11 samples presented conflicting results between the assays.

Thus, the respective sensitivity and specificity of iELISA were 92.31% and 91.67% compared with those of Western blot (Table 4). The total coincidence of iELISA and Western blot was 92.03% (Table 4). A κ coefficient of 0.838 (95% CI 0.735–0.925) was calculated between Western blot and iELISA. According to the criteria by Landis and Koch [11], this finding indicated an excellent agreement between iELISA and Western blot.

**Table 4:** Comparison of the ELISA and Western blot test

	Western blot results		
ELISA results	Positive	Negative	Total
Positive	72	5	77
Negative	6	55	61
Total	78	60	138
Total (% coincidence)	92.31(72/78)	91.67(55/60)	92.03(127/138)

### Application of iELISA for the detection of MiCV infection

**Fig. 4:**
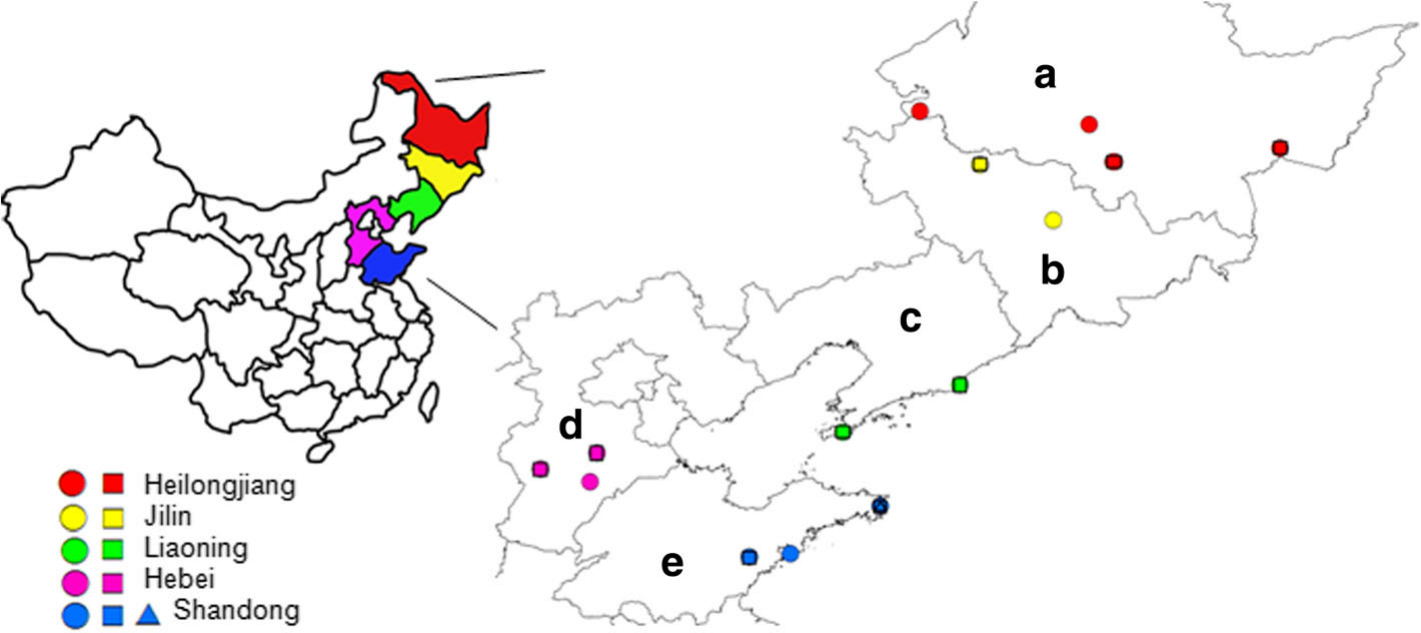
Geographical distribution of the sampled mink farms. The area covers 5 provinces in China: (**a**) Heilongjiang, (**b**) Jilin, (**c**) Liaoning, (**d**) Hebei, and (**e**) Shandong. The farms are marked with circles, squares, and triangles

To evaluate iELISA, we detected the antibodies against MiCV in the sera obtained from the minks from Shandong, Hebei, Heilongjiang, Jilin, Liaoning provinces in China by using the proposed technique (Fig. 4). Our results are shown in Table 5. Overall, the positive rate of MiCV-specific antibodies was 23.87% (163/683) in mink farms, and these sera were found positive by iELISA with an OD range of 0.313–2.256 (mean 0.413; S.D. 0.111). Of these, 21 of 24 farms with 163 of 683 (23.87%) individuals were tested positive for MiCV antibodies. The positive rates for each of the 21 flocks ranged from 2.33% to 73.68% (Table 5). The positive rates of MiCV in different mink farms were 13.04% in Heilongjiang Province, 27.87% in Jilin Province, 25.00% in Liaoning Province, 33.81% in Hebei Province, and 26.79% in Shandong Province (Table 5).

**Table 5:** Detection of MiCV-specific antibody in sera from breeder minks

Province	Farm	Number positive/number tested	Positive rate(%)
Heilongjiang	1	7/62	11.29
	2	9/31	29.03
	3	0/34	0
	4	6/23	47.83
	5	0/21	0
	6	5/36	13.89
Shandong	1	1/43	2.33
	2	13/29	44.83
	3	14/19	73.68
	4	11/31	35.48
	5	4/30	13.33
	6	2/16	12.50
Hebei	1	16/39	41.03
	2	6/33	18.18
	3	9/27	33.33
	4	14/22	63.64
	5	2/18	11.11
Liaoning	1	14/26	53.85
	2	0/17	0
	3	7/33	21.21
	4	6/32	18.75
Jilin	1	14/32	45.16
	2	1/12	8.33
	3	2/17	11.76
Total		163/683	23.87

**Fig. 5:**
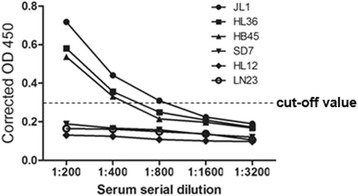
Anti-MiCV antibody titres in positive and negative mink serum samples. Positive and negative mink serum samples were prepared in dilutions of 1:200, 1:400, 1:800, 1:1600 and 1:3200 used in an ELISA assay. The cut-off value cutoff was determined by counting the mean OD value of the ELISA negative samples plus 3 standard deviation (SD)

Three positive samples and three negative samples were selected as examples to be used in iELISA to determine the titer for the serum samples. In Fig. 5, the antibody titer of the three positive (samples JL1, HL36 and HB45) samples was 1:800, 1:400 and 1:400, respectively, by the serial dilution of sera.

## Discussion

This study aimed to develop iELISA based on recombinant antigen for the detection of anti-MiCV antibodies in minks.

Cap is the only structural protein of circovirus and is the major immunogenic protein produced in circovirus-infected animals [12, 13]. Cap is the only structural protein of circovirus and is the major immunogenic protein produced in circovirus-infected animals. It had a large number of arginine residues concentrated at the N-terminus, which can inhibits the mRNA translation. As such, this protein is a major viral target, it plays a critical role in virus antigenic and useful tool for epidemiological surveys. Using recombinant capsid protein to develop ELISA method for the detection of circovirus like PCV2 and DuCV have been confirmed [9, 14–17]. Wang et al. reports that PCV2 can infect mink [18].

The amino acid sequences of Cap, especially in the first 40 amino acid residues of the N-terminal regions, in PCV2 are highly homologous to those of MiCV. To avoid the possible cross-reaction of the two viruses in future detections, we deleted the first 40 amino acid residues of the N-terminal regions, which unlikely affect protein antigenicity [9, 19].

In this study, the truncated Cap gene was cloned and the corresponding protein was expressed in *E. coli* because bacterial expression systems are more convenient. Using the purified recombinant Cap as a diagnostic antigen, we developed specific and sensitive iELISA to identify MiCV infection. Our data also demonstrated the specificity of this antigen, which does not react with antibodies directed against CAV-2, AMDV, MEV, PRV and CDV, *E. coli*, *P. multocida*, and *P. aeruginosa*.

In our experiments, goat anti-feline IgG secondary antibodies (Thermo Scientific) were selected and used as a secondary antibody. Checkboard ELISAs were also utilized to determine the following optimal working conditions: coating antigen concentration, 0.05 μg/ml; serum dilution, 1:200; detection antibody dilution, 1:20,000; blocking buffer, 5% skimmed milk; and incubation periods for the serum samples or the secondary antibodies, 90 and 60 min, respectively. The assay cut-off value based on the Western blot negative sera was defined as 0.08. After we compared the results of iELISA with those of Western blot, we found a coincidence of 83% between these methods. Therefore, we chose horseradish peroxidase-conjugated protein A/G as a detection reagent in rCap iELISA.

To date, no standard methods are available. As such, we compared the specificity and sensitivity of the newly developed iELISA in detecting MiCV-specific antibodies with Western blot. The data from rCap ELISA were consistent with the detection results from Western blot, with a relative sensitivity of 92.31% and a specificity of 91.67%. A kappa of 0.838 was statistically obtained to verify the concordance between iELISA and Western blot, resulting in an excellent agreement between these assays. According to the criteria by Landis and Koch [11], this finding suggested an excellent agreement between iELISA and Western blot.

We used the developed iELISA in this study to detect MiCV antibodies in clinical serum samples conducted in 24 farms and to understand the MiCV infection of large-scale mink farms in China.

The results indicated that most of the farms, except two farms, were MiCV seropositive. The total positive rate of MiCV antibodies was 23.87% in different minks. There are some differences in the prevalence of the MiCV infection in different provinces, and also obvious differences in the positive rate in different farms in the same area, such as the positive rate of MiCV antibodies in minks in Shandong was significantly higher than that of other minks in Heilongjiang.

Wang et al. applied PCR to detect 185 mink samples collected from Shandong, Hebei, and Liaoning provinces in September 2013 to September 2014 [8]. They obtained 101 positive samples, and the positive rate was 54.6%. The highest positive rate was 63.2% in Hebei, followed by 54% and 44.4% in Liaoning and Shandong, respectively.

These results suggested that MiCV infections are probably epidemic in Heilongjiang, Jilin, Shandong, Hebei, and Liaoning provinces in China. The harmful effects of this virus on mink farm production should be further investigated.

## Conclusions

MiCV infection has become common in major mink culture regions in China. rCap may be a useful antigen for the serodiagnosis of MiCV. iELISA with high levels of sensitivity, specificity, and reproducibility was developed successfully for the detection of antibodies to MiCV. The assay shows potential for the serological diagnosis of MiCV infection, large-scale serological surveys, and antibody titer monitoring.

## Abbreviations

AMDV: Mink aleutian disease virus
CAV-2: Canine adenovirus type 2
CDV: Canine distemper virus
CV: Coefficient of variation
iELISA: Indirect enzyme-linked immunosorbent assay
MEV: Mink enteritis virus
MiCV: Mink circovirus
ORF: Open reading frames
PCR: Polymerase chain reaction
PRV: Pseudorabies virus;
rCap: Recombinant capsid protein
SD: Standard deviation
