# Correction to: Development and application of an indirect enzyme-linked immunosorbent assay based on recombinant capsid protein for the detection of mink circovirus infection

**DOI:** 10.1186/s12917-018-1449-5

**Published:** 2018-04-10

**Authors:** J. Ge, X. Cui, Y. Shi, L. Zhao, C. Wei, S. Wen, S. Xia, H. Chen

**Affiliations:** 1 College of Veterinary Medicine, Northeast Agricultural University, Harbin, 150030 China; 2 Northeastern Science Inspection Station, China Ministry of Agriculture Key Laboratory of Animal Pathogen Biology, Harbin, 150030 China; 3 Laboratory Animal and Comparative Medicine Unit, Harbin Veterinary Research Institute, The Chinese Academy of Agricultural Sciences, No. 678 Haping Rd, Harbin, 150069 China; 4 Laboratory Animal and Comparative Medicine Unit, Harbin Veterinary Research Institute, The Chinese Academy of Agricultural Sciences, No. 678 Haping Rd, Harbin, 150069 China

## Correction

The original article [1] contained an error whereby Fig. 2 had processed incorrectly. The correct version of Fig. 2 is now displayed in the original article as well as ahead.

**Fig. 2:**
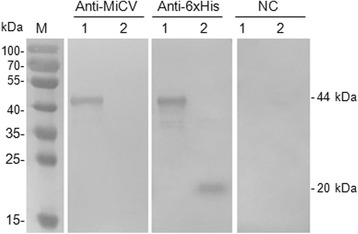
Western blot was performed with MiCV positive sera (Anti-MiCV), Anti-6xHis HRP conjugated (Anti-6xHis) or MiCV negative sera control (NC). Lane M: protein molecular weight marker; Lane 1: Purified protein rCap; Lane 2: pET32a vector control
